# In neonatal sepsis every catheter matters

**DOI:** 10.1038/s41390-021-01533-3

**Published:** 2021-04-17

**Authors:** Andreas Ohlin, Louise Björkman Hjalmarsson

**Affiliations:** 1 Department of Pediatrics, Faculty of Medicine and Health, Örebro University, Örebro, Sweden; 2 Department of Pediatrics, Örebro University Hospital, Örebro, Sweden

We would like to thank and congratulate Letouzey and co-workers for writing a very interesting article on the background factors that causes late-onset sepsis (LOS) in very preterm infants born before 32 weeks.^1^ The article is based on the large French cohort called EPIPAGE-2, which includes 4227 live births. The 2052 singletons alive at 72 h constitute the study population for the work presented in the February issue of *Pediatric Research*.^2^ Of the included infants, 437 contracted LOS, and with such a large material, the authors took on the challenging task to try to sort out if the cause of the preterm birth itself is a risk factor for LOS.

One of the obvious challenges in a study like this is that many of the risk factors are so closely linked together: Cause for prematurity → fetal growth restriction → parenteral nutrition → intravascular catheter → LOS. It is, therefore, extremely difficult to be sure in which of these steps the major risk factor is located and the fact that Letouzey et al. has accepted this challenge and given us some new insights into this spider web of risk factors is admirable. According to their article, fetal growth restriction and hypertensive disorders are strong risk factors irrespectively of the duration of the central catheter. This is a somewhat surprising conclusion, especially since the majority (66%) of LOS cases in the cohort were caused by *coagulase-negative staphylococci* (CoNS). CoNS is a pathogen well known for its strong tendency to attach to foreign materials and thereby is a common cause of catheter-related infections.^3,4^ As the authors claim, it is true that CoNS have been found in the intestinal tract of infected newborns but that does not mean that this is the port of entry for the identified bacteria.

The association between infection and intravascular catheters has been described in numerous well-conducted studies and in meta-analysis.^5,6^ Different sorts of intravascular catheters, such as umbilical lines, central vascular catheters, peripherally inserted central lines, and peripheral venous catheters, have all been described as important risk factors for LOS.^7–10^ In addition, it has been repeatedly shown that LOS in general and especially sepsis caused by CoNS can be prevented by implementing care bundles focused on improving the hygiene routines concerning intravenous (i.v.) lines.^11–17^ This strongly suggests that a considerable proportion of CONS sepsis cases is caused by a contaminated catheter.

With all this information in mind, it seems reasonable to collect very detailed data on i.v. lines when you try to distinguish antenatal risk factors from postnatal risk factors for LOS. We were, therefore, very surprised by how little data on i.v. lines that were presented in the Letouzey article. The article only explains that they collected information on the “duration of the central catheter”. Does this mean that only data on the first central catheter (umbilical) was collected? How were multiple catheters and multiple lumens handled, was each lumen considered as a separate line day? Were peripherally inserted central lines included in “the central catheter” or were they defined as peripheral? Do the authors have data on peripheral catheters?

We certainly understand the difficulties to collect such detailed data in a very large cohort, and if some of these data are missing, we would like to read the authors’ thoughts on how other types of catheters might be important for the conclusion.
